# Supplementary material for: Whole gut microbiome composition of damselfish and cardinalfish before and after reef settlement
Source: PeerJ. 2016 Aug 31;4:e2412. doi: 10.7717/peerj.2412 (PMC5012416; doi:10.7717/peerj.2412)
Supplement: Table S1 [file peerj-04-2412-s001.docx]

Table S1. Summary of all fish samples collected/analyzed

Family Genus Species Life Stage No. of sequences OTUs post rarefaction Fish Length (mm)

Pomacentridae Pomacentrus moluccensis Pre-settlement 9848 102 13

Pomacentridae Pomacentrus moluccensis Pre-settlement 4932 155 12

Pomacentridae Pomacentrus moluccensis Pre-settlement 7610 557 11

Pomacentridae Pomacentrus moluccensis Pre-settlement 9197 250 11

Pomacentridae Pomacentrus chrysurus Pre-settlement 64390 148 15

Pomacentridae Pomacentrus chrysurus Pre-settlement 19825 135 14

Pomacentridae Pomacentrus chrysurus Pre-settlement 22546 107 14

Pomacentridae Pomacentrus chrysurus Pre-settlement 10228 170 14

Pomacentridae Pomacentrus nagasakiensis Pre-settlement 20696 61 16

Pomacentridae Pomacentrus nagasakiensis Pre-settlement 28458 107 16

Pomacentridae Pomacentrus nagasakiensis Pre-settlement 11828 110 15

Pomacentridae Pomacentrus nagasakiensis Pre-settlement 34479 69 16

Pomacentridae Pomacentrus amboinensis Pre-settlement 5887 216 14

Pomacentridae Pomacentrus amboinensis Pre-settlement 10060 253 14

Pomacentridae Pomacentrus amboinensis Pre-settlement 74686 131 13

Pomacentridae Pomacentrus amboinensis Pre-settlement 25807 303 16

Apogonidae unknown unknown Pre-settlement 13096 158 8

Apogonidae unknown unknown Pre-settlement 80429 106 8

Apogonidae unknown unknown Pre-settlement 10393 139 8

Apogonidae unknown unknown Pre-settlement 31293 164 9

Apogonidae unknown unknown Pre-settlement 36882 375 8

Apogonidae unknown unknown Pre-settlement 22128 212 10

Apogonidae unknown unknown Pre-settlement 30059 226 10

Pomacentridae Pomacentrus wardii Pre-settlement 38610 251 14

Pomacentridae Pomacentrus wardii Pre-settlement 82997 258 15

Pomacentridae Pomacentrus wardii Pre-settlement 90795 141 15

Pomacentridae Pomacentrus wardii Pre-settlement 138124 311 16

Pomacentridae Pomacentrus bankanensis Pre-settlement 12279 63 13

Pomacentridae Pomacentrus bankanensis Pre-settlement 8732 145 13

Pomacentridae Pomacentrus bankanensis Pre-settlement 12333 164 14

Pomacentridae Pomacentrus bankanensis Pre-settlement 7197 104 11

Pomacentridae Pomacentrus coelestis Pre-settlement 22552 267 15

Pomacentridae Pomacentrus coelestis Pre-settlement 7089 257 15

Pomacentridae Pomacentrus coelestis Pre-settlement 8211 124 15

Pomacentridae Pomacentrus coelestis Pre-settlement 25037 244 15

Pomacentridae Dascyllus aruanus Pre-settlement 4398 178 9

Pomacentridae Dascyllus aruanus Pre-settlement 16510 278 9

Pomacentridae Dascyllus aruanus Pre-settlement 6478 180 9

Pomacentridae Chromis unknown Pre-settlement 4440 171 11

Pomacentridae Chromis unknown Pre-settlement 5366 193 12

Pomacentridae Chromis unknown Pre-settlement 8756 178 11

Pomacentridae Chromis unknown Pre-settlement 12667 187 11

Pomacentridae Chromis unknown Pre-settlement 10637 99 11

Pomacentridae Chromis unknown Pre-settlement 42028 148 11

Pomacentridae Chromis unknown Pre-settlement 8226 138 11

Apogonidae Ostorhinchus doederleini Pre-settlement 59740 155 12

Apogonidae Ostorhinchus doederleini Pre-settlement 13338 55 11

Apogonidae Ostorhinchus doederleini Pre-settlement 5921 206 12

Apogonidae Ostorhinchus doederleini Pre-settlement 6887 173 12

Pomacentridae Dascyllus aruanus Post-settlement 31404 163 30

Pomacentridae Dascyllus aruanus Post-settlement 4262 258 24

Apogonidae Ostorhinchus doederleini Post-settlement 33602 297 29

Apogonidae Ostorhinchus doederleini Post-settlement 16642 162 28

Apogonidae Ostorhinchus doederleini Post-settlement 10612 287 32

Apogonidae Ostorhinchus doederleini Post-settlement 56229 294 33

Pomacentridae Pomacentrus moluccensis Post-settlement 29632 320 36

Pomacentridae Pomacentrus moluccensis Post-settlement 71204 93 31

Pomacentridae Pomacentrus moluccensis Post-settlement 8672 181 29

Pomacentridae Pomacentrus moluccensis Post-settlement 26880 187 34

Pomacentridae Pomacentrus chrysurus Post-settlement 18609 44 25

Pomacentridae Pomacentrus chrysurus Post-settlement 30552 183 27

Pomacentridae Pomacentrus chrysurus Post-settlement 54271 49 27

Pomacentridae Acanthochromis polyacanthus Post-settlement 16725 422 14

Pomacentridae Acanthochromis polyacanthus Post-settlement 5293 325 16

Pomacentridae Acanthochromis polyacanthus Post-settlement 10162 351 15

Pomacentridae Pomacentrus moluccensis Post-settlement 15679 228 24

Pomacentridae Pomacentrus moluccensis Post-settlement 11487 124 26

Pomacentridae Chromis unknown Post-settlement 18184 299 31

Pomacentridae Chromis unknown Post-settlement 30348 318 25

Pomacentridae Chromis unknown Post-settlement 25948 301 22

Pomacentridae Dascyllus aruanus Post-settlement 27731 322 25

Pomacentridae Dascyllus aruanus Post-settlement 18668 281 27

Pomacentridae Dascyllus aruanus Post-settlement 45540 380 30
